# Nurses’ core disaster-response competencies for combating COVID-19—A cross-sectional study

**DOI:** 10.1371/journal.pone.0252934

**Published:** 2021-06-10

**Authors:** Igor Karnjuš, Mirko Prosen, Sabina Ličen

**Affiliations:** Faculty of Health Sciences, University of Primorska, Izola, Slovenia; University of Botswana Faculty of Medicine, BOTSWANA

## Abstract

The core competencies in disaster nursing, nurses’ roles in disaster management and the potential barriers are assessed with a view to developing disaster nursing in Slovenia. Despite training and experience, many indicators show nurses are deficient in skills involving emergency and disaster-preparedness competencies. Nurses report little familiarity with disaster-planning skills, the implementation of disaster guidelines and assessment of patients subject to a disaster circumstance. A cross-sectional descriptive study was conducted based on data collected through an online survey. 118 registered nurses from different clinical settings in Slovenia participated in the study. Data were collected according to the Slovenian version of the Disaster Nursing Core Competencies Scale (Sl-DNCC-Scale). The scale was limited to a 7-point Likert response format (from 1 = strongly disagree to 7 = strongly agree). The results show the registered nurses perceive the core competencies of disaster nursing to be important to their preparedness for disaster situations (median = 161; range 74–189). Registered nurses who work in nursing homes and nurse managers are more aware of the importance of acquiring the listed competencies for unexpected events (p = 0.011 and 0.060 respectively) and the importance of their active role in disaster management (p = 0.027 and p = 0.004, respectively). To effectively deal with a disaster, nurses must be well prepared for unexpected events and more actively involved in disaster management. This study demonstrates that nurses regard the core disaster nursing competencies as important and greatly needed in various healthcare facilities. Future studies in this area should focus on ways to implement these competencies in nursing education.

## Introduction

In recent years, the number of both natural and man-made disasters has grown and, wherever they occur, they have significant impacts on health, bringing human suffering and disrupting communities [1–3]. Disasters are typically characterised by their unpredictable, unexpected, uncertain and unplanned nature [1]. Disasters create special circumstances that require healthcare professionals to adapt to the changed environment and working conditions. As the largest group of healthcare professionals, nurses play crucial roles while responding to emergency events. In fact, from a disaster management perspective, nurses are usually the first health professionals to meet patients during a disaster event. Throughout the history of disasters, regardless of the disaster type, nurses have always been involved in every phase of disaster management, including mitigation, preparedness, response and recovery [1, 4, 5].

Currently, the whole world is in a state of emergency due to the COVID -19 pandemic (a completely new coronavirus called SARS-CoV-2) that has caused deaths, disabilities and economic crises [6]. The extremely high transmissibility of the virus saw the disease begin to spread uncontrolled around the world. In a situation like this, it is essential to protect the population’s health. In this respect, different professional groups (e.g. healthcare workers such as emergency and paramedical teams, epidemiologic services and the Civil Protection organisation) are the first to intervene [7]. Nurses make up the largest share of healthcare workers and have always played a considerable role in all phases of disaster management [8, 9]. However, more recent literature emphasises that nurses are still insufficiently prepared to respond to disasters [2, 4, 10].

It is vital that healthcare workers are prepared for disasters so that they can protect themselves and the community in the event of a disaster. Recently published data reveal that not only civilians, but also many healthcare workers around the world have died unnecessarily from COVID-19 [11]. Several authors have attempted to summarise the core nursing competencies in the case of a disaster, yet there is still no consensus on what these competencies are [1, 12]. Like in many other countries, the core disaster management competencies that nurses need are not yet clearly defined and hence not recognised in Slovenia. Therefore, an appropriate management strategy must be applied in nursing practice to reduce and control the negative effects of disasters [8].

The first confirmed case of COVID-19 in Slovenia was announced on 4 March 2020. On 11 March 2020, the WHO declared a COVID-19 pandemic and 1 day later the Government of the Republic of Slovenia declared an epidemic, activating the National Disaster Preparedness Plan [13–16]. The entire world is today facing one of the largest pandemics in its history due to COVID-19. Between 30 December 2019 and 26 December 2020, 79.062,802 cases of COVID-19 were identified worldwide, of whom 1.751,311 died [17]. By 26 December 2020, 114,729 people in Slovenia had tested positive (of the total number of people tested by then of 676,723), of whom 2,573 died [15], with current data still not indicating any easing of the epidemic. In fact, the number has been rising again in recent months in several European countries.

There is a formal global consensus that nurses require some knowledge of disaster management, leading to the development of the disaster nursing concept [18] that stresses the need to improve nurses competencies in such situations. Indeed, in a disaster, nurses may be involved in various aspects of disaster management as coordinators, information distributors, emotional and psychological supporters, and clinical and first-aid providers. They can also triage victims and prioritise patients’ care needs [8, 19, 20].

Research shows the present education system and training do not provide the necessary skills and that nurses feel poorly prepared for disaster response [2, 3, 6, 8]. In fact, most nurses do not encounter disaster situations very much and thus lack experience and opportunities to develop their expertise [8]. Moreover, disaster nursing is a relatively new specialty in the early stages of development worldwide, while the contents of disaster nursing are still not fully integrated into nursing curricula [4]. A large number of competencies related to disaster nursing have been developed, although evidence is lacking as to the most appropriate set of these core competencies. In 2019, the International Council of Nurses (ICN) published a new revised Framework of Disaster Nursing Competencies outlining eight domains of nurse competence in disaster situations: preparation and planning, communication, incident management systems, safety and security, assessment, intervention, recovery, and law and ethics [21]. This framework describes what a nurse should be capable of doing in a given situation, depending on their professional expertise.

Over the past decade, there has been a growing awareness in Slovenia at the national level of the importance of properly prepared nurses and other health professionals, especially in the fields of trauma, hospital and prehospital emergency care, for organising adequate training through licensed disaster drills and for providing disaster management courses [22]. In 2013, the Ministry of Health of the Republic of Slovenia also developed operating guidelines for the medical emergency system in the event of mass casualties [23], although the document only defines certain tasks that nurses perform in the case of mass casualties. The current legislation does not define the competencies of nurses for responding to a disaster. This raises the question of whether nurses in Slovenia consider themselves as prepared to manage disasters in the region.

Due to their different experiences and qualifications, nurses may possess a range of knowledge and skills concerning disaster preparedness. However, the problems this creates can be addressed by identifying gaps in their expertise and then ensuring further education and training.

## Methods and methods

### Ethical considerations

The study was conducted according to ethical principles [24]. All data were treated with confidentiality. The study was granted permission from the Commission for Scientific Research Work at the University of Primorska.

### Aim

To explore how registered nurses perceive the core competencies entailed in disaster nursing, their role in disaster management, and the potential barriers with a view to developing disaster nursing in Slovenia. In addition, we examine the relationship between the socio-demographic characteristics of registered nurses in relation to the three aforementioned domains.

### Design

The study had a cross-sectional design. A questionnaire based on The Slovenian version of the Disaster Nursing Core Competencies Scale was used to collect the data. The questionnaire was answered by registered nurses (RNs) from different clinical facilities in Slovenia.

### Participants

A convenience sample of RNs was obtained from 402 graduate nursing students who had completed the 1st cycle of the undergraduate nursing education program within the past five years and are currently working in clinical settings. Given that the research was conducted during the SARS-CoV-2 pandemic, we asked former nursing students working in clinical practice to share the link to the online survey with their colleagues. Their data and contacts were recovered from the faculty register. Although the calculated sample size based on confidence level 95% with a 5% margin of error is 367, we were unable to access clinical practice in any other way due to the situation at the time. The study was conducted between April and May 2020. RNs received an email invitation explaining the study’s purpose, assuring that participation was voluntary and there would be no consequences for opting out or withdrawing from the study.

### Instrument

Data were collected based on the Disaster Nursing Core Competencies Scale [4]. The questionnaire was designed to develop a valid and reliable scale that identifies and explores the core competencies of disaster nursing, nurses’ roles in disaster management, and the potential barriers in this regard. The Disaster Nursing Core Competencies Scale initially consisted of 43 items and 3 subscales: (i) Nurses’ core competencies in disaster management (30 items); (ii) Barriers to developing core competencies (8 items); and (iii) Nurses’ roles and responsibility in disaster management (5 items) [4].

The questionnaire and permission to use it for research purposes were obtained from the lead author Abdulellah Al Thobaity [4]. The original questionnaire was translated from English into the Slovenian language independently by the two authors who have disaster nursing experience. Further, a translation-back translation was performed to test the consistency of the Slovenian version against the original text. In addition, the required psychometric testing was conducted, which included measuring face validity, content validity, construct validity, and internal consistency, including confirmatory factor analysis. Factor analysis provided a three-factor model with the first factor Nurses’ core competencies in disaster management explaining 36.53% of the total variance and total. The results also showed a very high correlation between factors (p < .001) and high reliability of the translated scale (Cronbach α = .937) [16]. The Slovenian version of the Disaster Nursing Core Competencies Scale or Sl-DNCC-Scale (*Slo*. Lestvica profesionalnih kompetenc medicinskih sester v izrednih razmerah or LPKMS-IR) consists of 39 items (score 39 to 273) and 3 subscales: (i) Nurses’ core competencies in disaster management (27 items; score 27 to 189); (ii) Barriers to developing core competencies (8 items; score 8 to 56); and (iii) Nurses’ roles and responsibility in disaster management (4 items; score 4 to 28) [16]. For research purposes, the scale was limited to a 7-point Likert response format (1 = strongly disagree, 4 = no opinion, 7 = strongly agree). Lower scores indicate lower perceptions of existing nursing core competencies in disaster management, lower agreement with the presence of barriers to developing core competencies, and a reduced perception regarding the role and responsibilities in disaster management and higher scores indicate the opposite.

### Data collection

RNs consented to participate in the study by clicking on an embedded link and completing the electronic survey (1ka.si; https://www.1ka.si/d/en). Again, appropriate written information about the study’s aim and their rights as participants was added to the questionnaire. RNs had an opportunity to complete the survey within 1 month, from April to May 2020. Survey reminders followed 2 weeks after the invitation had been distributed. The respondents’ answers to the questions were collected in a database on the 1ka.si webserver. At the end of the survey, all of the data were simultaneously transferred, meaning that any data for still uncompleted surveys were not saved. In the data collection process, a database was created that included respondents’ answers without their names, surnames and e-mail addresses to ensure anonymity. The data collection was the sole responsibility of one researcher and was maintained in a password-secured 1ka.si account. For statistical analysis purposes, the data were then exported to SPSS.

### Data analysis

The IBM Statistics Package for Social Sciences (SPSS) Version 25 for macOS was used to analyse the data. Descriptive statistics like frequencies, percentages, means, medians and standard deviations were used to describe and summarise. Since the data were not normally distributed (Kolmogorov-Smirnov test p < .0001), both the non-parametric Kruskal Wallis H test and the Mann-Whitney U test were used to establish statistically significant differences between the groups. In addition, a Pearson’s chi-squared test was used to test relationships between categorical variables. A p-value ≤ .05 was considered significant.

## Results

Of the 402 RNs invited, an additional 36 RNs participated in the study based on a snowball sampling. A total of 118 participants completed the questionnaire. Since participation in the study was voluntary, the overall response rate for registered nurses was 26.9%. The RNs consisted of 83.1% women (n = 98) and 16.9% men (n = 20). In addition, our sample (n = 118) represents 1.9% of all RNs currently employed in Slovenia (N = 6.249). The participants’ average age is 36 years (SD = ± 10.6). Sample characteristics are shown in Table 1.

**Table 1:** Demographic data concerning the participants.

Variables (n = 118)	n	%
Gender		
Male	20	16.9
Female	98	83.1
Health facilities		
Primary Health Care	56	47.5
Secondary Health Care	32	27.1
Tertiary Health Care	16	13.6
Social Welfare Institutions	12	10.2
No response	2	1.6
Nurse manager		
Yes	23	19.5
No	92	78.0
No response	3	2.5

Note. RNs–Registered nurses

To examine how RNs perceive the entailed core competencies and their role and responsibilities in disaster management, and the related potential barriers to developing core competencies for disaster nursing, the Sl-DNCC-Scale was used. In line with the aim, the first set of results is based on descriptive statistics (min, max, mean and standard deviation) and on Sl-DNCC-Scale scoring. The Sl-DNCC-Scale score by the RNs was (mean = 5.54, median = 5.56, IQR = 1, SD = .696 [95% CI 5.41, 5.67], p = .000). Table 2 presents the respondents’ descriptive statistics for each item of the Sl-DNCC-Scale.

**Table 2:** Sl-Disaster Nursing Core Competencies Scale (n = 118)–descriptive statistics.

Items	Min	Max	Mean	SD
Subscale 1 “Nurses’ core competencies in disaster management”				
*A nurse should be able to*				
demonstrate an ability to follow and work within an incident management system.	1	7	5.98	1.177
identify potential threats with medical implications in response areas.	2	7	5.76	1.243
provide defensible solutions to a series of ethical dilemmas arising in disaster.	1	7	5.60	1.289
understand the purpose of disaster plan.	1	7	6.14	1.124
describing the principles of crisis communication in crisis intervention and risk management.	1	7	5.82	1.264
maintain knowledge in areas relevant to disaster and disaster nursing.	1	7	6.12	1.262
describe nurses roles in various disaster assignments (e.g. shelters, emergency care sites).	2	7	5.62	1.251
participate in planning to meet health care needs in a disaster.	2	7	6.00	1.094
recognise the disaster plan in the workplace and ones role in the workplace at the time of a disaster.	1	7	6.19	.972
describe strategies for allocating scarce resources in an ethical manner to optimise population outcomes during triage and treatment.	2	7	5.40	1.300
list the appropriate steps for requesting psychological first aid for responders, patients, and other victims.	1	7	5.45	1.270
understand how to prioritise care and manage multiple situations.	2	7	6.14	.981
manage the resources and supplies required to provide care in the community.	1	7	5.31	1.417
identify vulnerable populations and coordinating activities to reduce risk.	3	7	6.11	.908
participate in creating new guidelines for nursing practice in disaster.	2	7	5.86	1.276
describe the phases of the disaster management continuum: prevention/mitigation, preparedness, response and recovery/rehabilitation.	2	7	5.62	1.116
provide up-to-date information to the disaster response team regarding health care issues and resource needs.	1	7	5.74	1.143
develop and maintain a personal and family preparedness plan.	1	7	5.70	1.200
understand the component of disaster plan.	1	7	6.01	1.195
use recordkeeping processes to ensure continuity of patient information.	2	7	5.78	1.118
list and apply principles for managing patients with the most common victims presentations, e.g., environmental illnesses; burns; blast and crush injuries; nuclear, biologic and chemical	1	7	5.95	1.246
facilitate and perform patient transport effectively and safely during a disaster.	1	7	6.14	1.127
participate in drills in the workplace and community.	1	7	6.05	1.305
participate in processes of securing adequate personal, supplies, equipment, and space for patient care (surge capacity).	1	7	5.77	1.355
identify and communicate important information immediately to appropriate authorities.	1	7	6.11	1.028
understand relevant disaster terminology.	3	7	6.05	.895
prioritise patients to maximise survivability.	3	7	6.22	.853
Subscale 2 “Barriers to developing core competencies”				
Lack of training programs in the workplace.	2	7	6.06	1.172
Ineffective training opportunities.	1	7	6.02	1.281
Lack of formal educational resources.	1	7	6.00	1.169
Lack of health organisation support.	2	7	5.88	1.347
Lack of expert staff in disaster nursing.	2	7	6.25	1.141
Lack of evaluation instruments.	1	7	5.94	1.270
Restriction of nurses roles in disaster management.	1	7	5.86	1.432
Lack of research studies on disaster nursing.	2	7	6.04	1.243
Subscale 3 “Nurses’ roles and responsibility in disaster management”				
I participate in education and training activities for health care providers that relate to disaster management.	1	7	3.00	2.172
I have a role in identifying the education and training needs of nurses with regard to disaster management.	1	7	3.02	2.034
I am authorised to activate a local disaster plan.	1	7	2.62	1.974
I participate in developing disaster plans and policy in my hospital.	1	7	3.23	2.213

The highest-rated items from the first subscale, measuring the perception on disaster core competencies were that “A nurse should be able to prioritise patients to maximise survivability” and “A nurse should be able to recognise the disaster plan in the workplace and one’s role in the workplace at the time of a disaster”. In addition, the study participants agreed that there are many barriers associated with developing the core competencies for disaster nursing, such as “Lack of expert staff in disaster nursing” and “Lack of training programmes in the workplace”. The RNs also agreed that their role and responsibilities in disaster management is low and rated these items lowest “I am authorised to activate a local disaster plan” and “I participate in education and training activities for healthcare providers that relate to disaster management”.

The sum score was high and exceeded the calculated median for the entire Sl-DNCC-Scale (median = 218, IQR = 33, [95% CI 212.90, 223.0], p = .000). Results also indicate that RNs perceive the related core competencies to be important in disaster nursing, rating the first subscale higher than determined by the median (median = 161.50, IQR = 29, [95% CI 153.77, 162.37], p = .000). RNs also agreed that listed barriers associated with the further development of disaster nursing exist (median = 50, IQR = 11, [95% CI 46.60, 49.52], p = .000) and that their role and responsibilities in disaster management as nurses is minor (median = 9, IQR = 12, [95% CI 10.54, 13.19], p = .000).

In order to examine the relationship between the socio-demographic characteristics of RNs in relation to the Sl-DNCC-Scale and the three subscales, the Mann-Whitney U test, Kruskal-Wallis H test, and Pearson Chi-Square test were conducted (Table 3).

**Table 3:** Demographic characteristics of the sample population concerning the Sl-DNCC-Scale and the three subscales.

Variable	1^st^ subscale	2^nd^ subscale	3^rd^ subscale	Sl-DNCC-Scale
	M (95% confidence interval)			
	*Test value/p*			
Gender				
Male	5.87 (5.52–6.05)	6.13 (5.33–6.42)	2.88 (2.35–3.82)	5.50 (5.25–5.71)
Female	6.00 (5.69–6.05)	6.25 (5.84–6.23)	2.00 (2.57–3.32)	5.57 (5.41–5.70)
	*(U)*856.000 /	*(U)*898.500 /	*(U)*846.500 /	*(U)*899.000 /
	.*373*	.*557*	.*336*	.*561*
Health facilities				
Primary Health Care	6.00 (5.56–6.02)	6.00 (5.61–6.16)	2.50 (2.57–3.52)	5.56 (5.31–5.66)
Secondary Health Care	5.67 (5.36–6.02)	6.44 (5.96–6.52)	1.88 (1.76–2.99)	5.44 (5.15–5.67)
Tertiary Health Care	6.15 (5.54–6.40)	6.25 (5.75–6.61)	2.38 (2.09–3.75)	5.65 (5.28–6.01)
Social Welfare Institution	6.54 (6.18–6.84)	6.50 (5.42–6.77)	4.50 (2.91–5.68)	6.05 (5.72–6.54)
	*(H)*11.062/	*(H)*2.954/	*(H)*9.201/	*(H)*8.669/
	.*011*	.*399*	.*027*	.*034*
Active working years as RNs				
From 1 to 7	5.96 (5.57–5.99)	6.13 (5.75–6.22)	2.25 (2.32–3.20)	5.56 (5.29–5.63)
From 8 to 14	6.09 (5.50–6.35)	6.44 (5.50–6.64)	1.88 (1.85–3.65)	5.62 (5.29–5.91)
From 15 to 22	5.56 (5.34–5.90)	6.19 (4.89–6.69)	2.75 (1.86–3.31)	5.40 (4.96–5.63)
From 23 to 30	6.17 (5.34–6.45)	6.19 (5.60–6.53)	2.38 (2.27–4.36)	5.71 (5.23–6.00)
From 31 to 39	6.52 (5.62–6.79)	6.38 (5.56–6.67)	3.50 (2.62–5.28)	5.86 (5.35–6.45)
	*(H)*9.044/	*(H)*1.216/	*(H)*3.789/	*(H)*6.690/
	.*060*	.*875*	.*435*	.*153*
Nurse manager				
Yes	6.30 (5.70–6.50)	6.63 (5.78–6.61)	4.50 (3.64–5.17)	5.86 (5.58–6.21)
No	5.93 (5.64–5.96)	6.25 (5.76–6.17)	2.00 (2.23–2.91)	5.50 (5.32–5.58)
	*(χ2)*89.199/	*(χ2)*43.658/	*(χ2)*72.998/	*(χ2)*173.473/
	.*945*	.*651*	.*004*	.*048*

Note. Nurses’ core competencies in disaster management–First subscale; Barriers to developing core competencies–Second subscale Nurses’ roles and responsibility in disaster management–Third subscale; Median (M); U value—Mann-Whitney U test; χ2 value—Kruskal Wallis H test; χ2 value—Pearson chi-square test; p value—statistical significance

Although the results reveal some differences between the groups, in relation to gender and active working years as RN the results are not statistically significant (p >.05). Further, the results show statistically significant differences (p < .05) when comparing the results between RNs working in different health facilities. The results reveal that those working in social welfare institutions assessed the subscales relating to Nurses’ core competencies in disaster management and Nurses’ roles and responsibility in disaster management, as well as the overall Sl-DNCC-Scale, higher than others working in primary, secondary and tertiary healthcare (median = 6.54; 95% CI = 6.18–6.84; median = 4.50; 95% CI 2.91–5.68; median = 6.05; 95% CI = 5.72–6.54, respectively).

Of all study participants, 19.5% held a leading position in relation to their workplace. Although they had higher scores for all three subscales and the entire Sl-DNCC-Scale than those who did not hold a leading position at work, there were no statistically significant differences between them (median = 6.30, 95% CI = 5.70–6.50; median = 6.63, 95% CI =. 5.78–6.61, respectively), except for the entire Sl-DNCC-Scale and the subscale Nurses’ roles and responsibility in disaster management (median = 5.86, 95% CI = 5.58–6.21; median = 4.50, 95% CI = 3.64–5.17, respectively) where the results show statistically significant differences (p < .05) between the groups.

## Discussion

A non-experimental, cross-sectional design was utilised to explore how RNs perceive the core competencies of disaster nursing, their role in disaster management, and the potential barriers among RNs from various clinical settings. The findings suggest that registered nurses perceive the core competencies of disaster nursing as important in their preparation for disaster situations. Registered nurses working in nursing homes and nurse managers are more aware of the importance of acquiring the listed competencies for unexpected events and the importance of their active role in disaster management.

This study shows that RNs agree that the core competencies involved in the first subscale of the Sl-DNCC-Scale are fundamental to their preparedness in disaster cases. The first subscale consists of many core competencies in disaster nursing such as planning, resources, triaging, communication, ethical issues, managing resources, psychological preparedness, workplace and community drills, and patient transportation [4]. Even though nurses are recognised as key players in disaster management, according to previous studies, they are often sub-optimally prepared for such tasks. This could pose a challenge at several levels among education providers and healthcare institutions in bridging the gaps in knowledge, skills and preparedness in disaster nursing management [8, 20, 25]. In Slovenia, some aspects of disaster preparedness are integrated into the nursing curriculum on the undergraduate level with a focus on the principles and management of patients in a disaster. However, while professional medical first-aid theories and skills, triage in the event of mass casualties are emphasized [23], specific educational needs such as psychological preparedness, assessment of disaster risks or comprehensive disaster response planning are often omitted. In fact most professional nurses retrieve this knowledge later in their careers through non-formal education, depending on the field in which they work, which is also a common practice in other countries around the globe [10]. The study also shows that RNs working in nursing homes gave stronger support for the subscales related to Nurses’ core competencies in disaster management than others working in primary, secondary and tertiary healthcare. Nurses working in nursing homes during the COVID-19 epidemic were some of the most challenged since their workplace, e.g. social welfare facilities, is not supposed to be a hospital-like environment yet the preventive strategy in the epidemic demanded the same (if not greater) preventive measures despite these facilities lacking preventive equipment and being understaffed [26, 27]. It is now clear that nursing homes with higher RN staffing and quality ratings hold the potential to better control the spread of the novel coronavirus and reduce related deaths [28].

The highest-rated subscale of the Sl-DNCC-Scale was the second one named Barriers to developing core competencies. Items within this subscale describe restricted roles of nurses and the lack of education, training, expertise, support and research and evaluation tools [4]. In this part of the questionnaire, there were no differences in the responses according to the socio-demographic characteristics of the sample. Our findings correspond somewhat with other studies that confirm that disaster nursing has unclear roles in the early stages and is missing adequate education, training, expertise and research [10, 19]. The main goal of education and training in disaster nursing is to physically and psychologically prepare nurses to respond to disasters and other crises [9, 29], not only in the immediate but also in the long term [8]. This can bring positive outcomes like a reduction of mortality in human populations, health promotion in the community and lower costs of healthcare organisations for people and communities in the country [20]. The results of this study on a sample of Slovenian nurses point out the need of nurses to receive additional and more focused education and training to prepare for disasters. The contents of this education and training should be planned and arranged for various disciplines with specific expertise, since they may not encounter the same similar problems or issues, as well as learning and teaching strategies that facilitate the development of critical-thinking skills and competence [9]. The latter should be aligned with the International Council of Nurses Framework of Disaster Nursing Competencies [21] because disasters are often not bound by country borders, making the international assistance of healthcare professionals, like during the COVID-19 crisis in the most-affected countries, particularly needed. Yet, the ICN document [21] describes three different levels of nurses expertise regarding competencies and disaster-preparedness skills, namely: (i) competencies are for all registered nurses; (ii) for nurses who are or aspire to be a designated disaster responder within an institution, organisation or system; and (iii) for nurses who respond to a wide range of disasters and emergencies. This means the same level of preparedness in disaster cases cannot be expected from all employed RNs [16].

Regarding the third subscale of the Sl-DNCC-Scale, Nurses’ roles and responsibility in disaster management, the items generally did not receive much support. The roles involve various aspects such as planning, education, training, drills and creating guidelines [4]. The study shows that RNs employed in nursing homes again gave this subscale more support than participants from other healthcare facilities. In fact, in Slovenia RNs in social welfare institutions are more involved in the development of disaster plans and policies in their facilities and have greater responsibility when the local disaster plan is activated, compared to healthcare institutions on primary, secondary, or tertiary level, where the primary role and greatest responsibility lies on the medical staff [23]. Furthermore, the combination of factors surrounding nursing care in nursing homes makes the COVID-19 pandemic a crisis for nursing homes in need of immediate policymaker and clinician support [26]. In this context, developing disaster-management skills of healthcare professionals as well as disaster plans specifically on coordinating care in nursing homes during disasters should become a priority. For example, the San Diego Disaster Preparedness Model for Nursing Homes has been used in the United States of America [30] and showed that this does not need require substantial resources from either nursing homes or other institutions and yet contributes greatly to the more effective coordination of care at a time of disaster.

Another aspect of the results is that nurses expressed the need to be included as key stakeholders in developing and planning disaster management system response. In particular, this aspect is observed in nurse managers who hold a leading position in the workplace as they agreed more strongly with all three subscales and the entire DNCC-Scale than other participants. A more visible role of nurses during the emergent phase of a disaster and throughout the disaster preparedness and recovery phases is in place when discussing the effective management of unpredictable events. According to Powers [31], nurses must be included in discussions concerning their community and healthcare disaster plans, whether regional or national, and their input should be mandated and integrated by emergency planners and healthcare leaders. The active inclusion of nursing insight and innovative thinking in disaster planning and preparedness will ensure that nursing care is provided to all who need it. Incorporating these would thus also contribute to the development of different approaches addressing the preparedness of nurses to respond to disasters.

### Strength and limitations

The study is the first of its kind in Slovenia, namely to explore the status and prospects of ability of disaster nursing competency. It was conducted at a time the COVID-19 epidemic had been declared in Slovenia and when nurses were challenged by new roles and responsibilities. Since disaster nursing is a new comprehensive cross-discipline [4, 32] and measurement instruments for exploring disaster nursing competency are scarce, the research findings may be of great interest to the professional community, especially in countries where disaster nursing is only starting as a new speciality within nursing.

Despite this contribution, this study has some limitations. The results should be interpreted with caution as only a small percentage of Slovenian registered nurses participated in it and thus the sample is not representative (32.15% of the optimal calculated sample size was achieved). In this context, it is also important to note the selection bias of the sample, as those who did not participate in the online survey may differ in their responses from those who did. Moreover, the settings in which the participants were employed were not equally distributed or sufficient to generalise the findings.

## Conclusion

RNs perceive the listed core competencies as fundamental to their disaster preparedness, yet they lack adequate education, training programmes, and expertise in disaster nursing. RNs who work in nursing homes and nurse managers who are in leadership positions are more aware of the active role of nurses and their responsibilities in disaster management. As the frequency and impact of disasters increase worldwide, there is a need to integrate disaster management into nursing education programmes, both in formal and continuing education. The core competencies of disaster nursing must be included in future research since the framework for disaster nursing competency is under the influence of various contextual factors that need to be investigated.

### Relevance to clinical practice

This study indicates that the core competencies in disaster nursing may serve as a basis for redesigning the curriculum for nursing faculties. The study results might be relevant to clinical leadership in advanced practice. Nurses need to become more aware of and understand their role in responding to disasters in order to contribute effectively to the team’s response. Future studies in this area should focus on how these competencies can be implemented in nursing education.

## Supporting information

S1 File (SAV)
